# Supplementary material for: Toward a Principled Workflow for Prevalence Mapping Using Household Survey Data
Source: J Surv Stat Methodol. 2026 Feb 6;14(1):209–37. doi: 10.1093/jssam/smaf048 (PMC13118171; doi:10.1093/jssam/smaf048)
Supplement: smaf048_Supplementary_Data [file smaf048_supplementary_data.pdf]

Reproducible report for 'Toward a Principled Workflow for Prevalence Mapping Using Household Survey Data'

2025-08-19

Contents

1 Overview **2**

2 Data Preparation **2**
2.1 Install ***surveyPrev*** and related R packages . . . 2
2.2 Process survey data . . . 3
2.3 Extract cluster location . . . 3
2.3.1 Checking the consistency of cluster locations . . . 4
2.3.2 Summarizing clusters in each area . . . 6
2.4 Extract area-level population information . . . 7
2.4.1 Population aggregating prevalence to upper level . . . 7
2.4.2 Urban/rural fraction for stratification model . . . 8
2.5 Cluster-level covariates . . . 9

3 Direct estimates **11**

4 Area-level (Fay-Herriot) Estimates **12**
4.1 Admin-1 Fay-Herriot estimates . . . 12
4.2 Admin-2 Fay-Herriot estimates . . . 13
4.3 Fay-Herriot model with area-level covariates . . . 14

5 Cluster-Level Estimates **15**
5.1 Urban/rural stratification . . . 15
5.2 Nested Admin-1 fixed effect . . . 18
5.2.1 Selecting fixed effect parameterization . . . 19
5.3 Using covariates . . . 20
5.3.1 Area-level covariates . . . 20
5.3.2 cluster-level covariates . . . 21

6 Visualization **24**

1 Overview

This reproducible report accompanies the paper *Toward a Principled Workflow for Prevalence Mapping Using Household Survey Data*. It demonstrates how prevalence mapping for binary indicators can be conducted using DHS data. All required datasets, including TIFF and CSV files, are available in the accompanying GitHub repository: https://github.com/dqianyu/WorkflowPaper.

In this report, we estimate the proportion of pregnant women who had at least four antenatal care visits using the 2022 Kenya DHS survey. Section 2 outlines the data preparation steps used to obtain and process the relevant sources. Sections 3 through 6 provide detailed instructions for fitting and visualizing the following 17 models using the **surveyPrev** package.

1. Admin-1 Direct estimates
2. Admin-2 Direct estimates
3. Admin-1 Fay-Herriot model
4. Admin-2 Fay-Herriot model
5. Admin-1 Fay-Herriot model with area-level covariates
6. Admin-2 Fay-Herriot model with area-level covariates
7. Admin-1 unstratified cluster-level model
8. Admin-2 unstratified cluster-level model
9. Admin-1 stratified cluster-level model
10. Admin-2 stratified cluster-level model
11. Admin-2 unstratified nested cluster-level model
12. Admin-2 stratified nested cluster-level model
13. Admin-2 stratified nested cluster-level model with intereaction
14. Admin-1 stratified cluster-level model with area-level covariates
15. Admin-2 stratified nested cluster-level model with area-level covariates
16. Admin-1 stratified cluster-level model with unit-level covariates
17. Admin-2 stratified nested cluster-level model unit area-level covariates

2 Data Preparation

2.1 Install *surveyPrev* and related R packages

The **surveyPrev** package can be installed from CRAN with

```
install.packages(surveyPrev)
```

The development version of the **surveyPrev** package can be installed from GitHub with

```
library(devtools)
install_github("richardli/surveyPrev")
```

After installation, the package can be loaded with

```
library(surveyPrev)
```

One of the key required packages, `INLA` is not available on CRAN or GitHub, and needs to be installed with the following code (Rue, Martino, and Chopin 2009).

```
install.packages("INLA", repos=c(getOption("repos"),
                 INLA="https://inla.r-inla-download.org/R/stable"), dep=TRUE)
library(INLA)
```

The following packages are needed to run the example analysis described in this report.

```
library(geodata)
library(sf)
```

```r
library(SUMMER)
library(survey)
library(rdhs)
library(ggplot2)
library(patchwork)
library(dplyr)
library(tidyr)
library(kableExtra)
library(terra)
library(raster)
```

2.2 Process survey data

Raw DHS data can be downloaded using `getDHSdata()` through the **rdhs** package.

```r
indicator <- "ancvisit4+"
year <- 2022
country <- "Kenya"
dhsData <- surveyPrev::getDHSdata(country = country, indicator = indicator, year = year)
```

Alternatively, if datasets have been downloaded from DHS website already, the data can also be manually loaded using scripts similar to the following.

```r
file_path <- "../KEIR8CDT/KEIR8CFL.DTA"
dhsData <- as.data.frame(haven::read_dta(file_path))
```

We then use `getDHSindicator()` to process the raw survey data into a data frame, where the column value is the indicator of interest. The data frame also contains information specifying survey designs in the `survey` package, including cluster ID, household ID, survey weight and strata information.

```r
data <-  surveyPrev::getDHSindicator(dhsData, indicator = indicator)
```

For this example, the data with anonymized household IDs can be find in Github.

```r
data <- read.csv("../WorkflowPaper/Data/anc_data.csv")
```

```r
head(data[,c("cluster", "householdID", "weight", "strata","value")])
```

```
##   cluster householdID  weight strata value
## 1       1           4 1296049  urban     1
## 2       1           7 1296049  urban    NA
## 3       1          10 1296049  urban    NA
## 4       1          13 1296049  urban    NA
## 5       1          20 1296049  urban    NA
## 6       1          26 1296049  urban    NA
```

2.3 Extract cluster location

We use `getDHSgeo()` function to download the GPS data for cluster locations thought the **rdhs** package. `geo` contains GPS coordinates that are used to match clusters to regions at different administrative levels.

```r
geo <- surveyPrev::getDHSgeo(country = country, year = year)
```

Similarly, it can also be performed manually by downloading the GPS file from the DHS website and read locally using `st_read`.

```r
file_path_geo<- "../KEGE8AFL/KEGE8AFL.shp"
geo <- sf::st_read(file_path_geo)
```

The Admin-1 and Admin-2 boundary shapefiles can be downloaded from the GADM site[1] and read into R manually. Another alternative is to use the **geodata** and **sf** package to download the maps from the GADM site and load them in R directly, in the following steps.

```
poly.adm1 <- geodata::gadm(country="KEN", level=1, path=tempdir())
poly.adm1 <- sf::st_as_sf(poly.adm1)
poly.adm2 <- geodata::gadm(country="KEN", level=2, path=tempdir())
poly.adm2 <- sf::st_as_sf(poly.adm2)
```

We use the `clusterInfo()` function to extract Admin-1 and Admin-2 area information for each cluster. To avoid confusion in countries with duplicated region names, we create new labels for Admin-2 regions using the variable `admin2.name.full`, which contains unique Admin-2 label in the format admin1.name_admin2.name. Clusters with invalid GPS locations are saved in the wrong.points object and are not used in any models.

```
cluster.info <- surveyPrev::clusterInfo(geo=geo,
                                       poly.adm1=poly.adm1,
                                       poly.adm2=poly.adm2)
```

2.3.1 Checking the consistency of cluster locations

DHS cluster locations are slightly jittered. This sometimes leads to mismatch between the recorded Admin-1 area and the assignment from cluster locations. DHS Admin-1 regions are usually recorded in the `v024` field, and below we conduct a quick check to see how many mismatches there are. The codes in this section is survey-specific and may not be needed for other surveys.

```
check <- left_join(data, cluster.info$data)
check <- check[match(unique(check$cluster), check$cluster), ]
assigned <- as.character(check$v024)
mapped <- tolower(check$admin1.name)
mapped[is.na(mapped)] <- "Not mapped"
mismatch <- check[mapped != assigned, ]
mismatch$assigned <- assigned[mapped != assigned]
mismatched_area <- (cbind(mapped[mapped != assigned],
                          assigned[mapped != assigned]))
colnames(mismatched_area) <- c("From GPS", "From DHS v024")
head(mismatched_area)
```

```
##      From GPS     From DHS v024
## [1,] "tana river" "garissa"
## [2,] "tana river" "garissa"
## [3,] "tana river" "garissa"
## [4,] "tana river" "garissa"
## [5,] "kirinyaga"  "tharaka-nithi"
## [6,] "kirinyaga"  "tharaka-nithi"
```

There are 20 clusters where the recorded Admin-1 area does not match what we obtain from GPS location. These require additional investigation of each cluster. We map each of these clusters to the closet Admin-2 region within the recorded Admin-1 region

```
maps <- NULL
new_admin <- data.frame(admin1.name = rep(NA, dim(mismatch)[1]),
            admin2.name = rep(NA, dim(mismatch)[1]),
            admin2.name.full = rep(NA, dim(mismatch)[1]))
for(i in 1:dim(mismatch)[1]){
  point <- subset(geo, DHSCLUST == mismatch[i, "cluster"])
  subpoly0 <- poly.adm2[tolower(poly.adm2$NAME_1) %in%
```

[1] https://gadm.org/download_country.html

```
                tolower(c(mismatch[i, c("admin1.name", "assigned")])), ]
  subpoly1 <- poly.adm2[tolower(poly.adm2$NAME_1) %in%
                tolower(c(mismatch[i, c("assigned")])), ]
  dist <- st_distance(point, subpoly1)
  new <- subpoly1$NAME_2[which.min(dist)]

  new_admin$admin1.name[i] <- subpoly1$NAME_1[which.min(dist)]
  new_admin$admin2.name[i] <- new
  new_admin$admin2.name.full[i] <- paste0(new_admin$admin1.name[i], "_",
                                          new_admin$admin2.name[i])

maps[[paste("ID", mismatch[i, "cluster"])]]<-
  mapPlot(data = NULL, geo = subpoly0,by.geo = "NAME_2") +
  geom_sf(data = subpoly1, color = "red", fill = NA, linewidth = 1.5) +
  geom_sf(data = subset(subpoly1, NAME_2 %in% new),
          color = "blue", fill = NA, linewidth = 1) +
  geom_point(data = point, aes(x = LONGNUM, y = LATNUM), size = 3) +
  ggtitle(mismatch[i, "cluster"])
}
```

As an example below, we plot the first three cluster locations. The black dots indicate the location of the cluster. The areas in red polygons are all the Admin-2 areas within the DHS recorded Admin-1 area. The area in blue polygon is the new Admin-2 area assignment after the correction. These are easier cases that can be corrected by visual inspection, as the clusters are very close to the boundary of the assigned Admin-1 area.

```
maps[["ID 209"]] + maps[["ID 210"]] + maps[["ID 216"]]
```

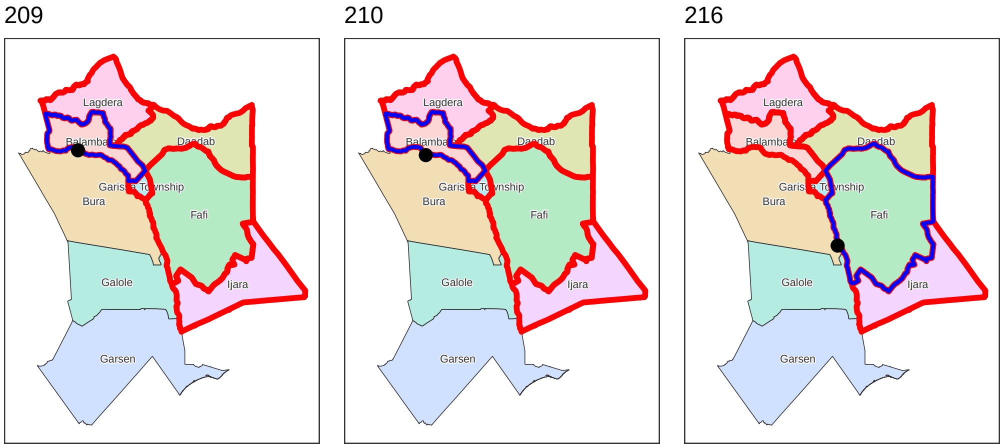

Figure 1: Examples of clusters that are mapped to the wrong Admin-1 area that can be corrected by visual inspection.

There are four clusters that are very far away from the recorded Admin-1 area. For these four clusters, a final determination requires additional information on which source of information is more trustworthy (GPS location or the v024 variable). We keep the area assignment from the GPS location in this analysis.

```
maps[["ID 1296"]] | maps[["ID 1297"]] | maps[["ID 1298"]] | maps[["ID 1299"]]
```

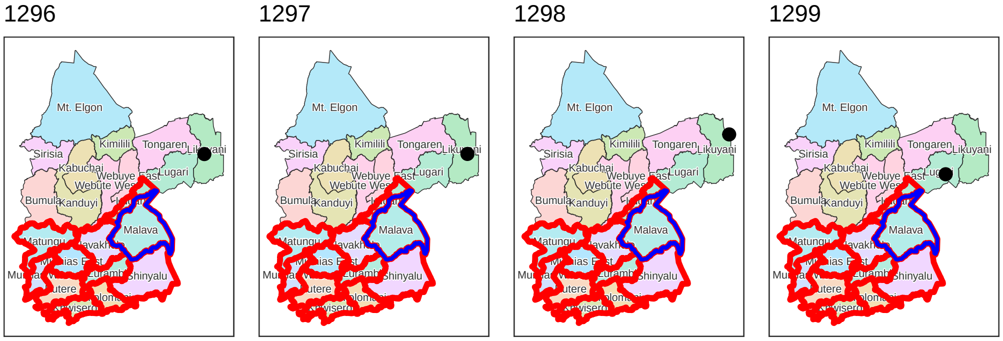

Figure 2: Examples of clusters that are mapped to the wrong Admin-1 area that are difficult to correct without additional information

We now correct the area assignment for clusters that are clear from visual inspection.

```r
for(i in 1:dim(mismatch)[1]){
  if(mismatch[i, "cluster"] %in% c(1296:1299)){
    next
  }
  sub <- which(cluster.info$data$cluster == mismatch[i, "cluster"])
  cluster.info$data[sub, "admin1.name"] <- new_admin[i, "admin1.name"]
  cluster.info$data[sub, "admin2.name"] <- new_admin[i, "admin2.name"]
  cluster.info$data[sub, "admin2.name.full"] <- new_admin[i, "admin2.name.full"]
}
```

2.3.2 Summarizing clusters in each area

The final cluster assignment are stored in the `cluster.info` object. There is one cluster without GPS location and are removed from analysis.

```r
head(cluster.info$data)
```

```
##   cluster  LONGNUM    LATNUM                   geometry admin1.name admin2.name
## 1       1 39.61278 -4.037417 POINT (39.61278 -4.037417)     Mombasa   Changamwe
## 2       2 39.62686 -4.033539 POINT (39.62686 -4.033539)     Mombasa   Changamwe
## 3       3 39.62328 -4.038505 POINT (39.62328 -4.038505)     Mombasa   Changamwe
## 4       4 39.61169 -4.035394 POINT (39.61169 -4.035394)     Mombasa   Changamwe
## 5       5 39.62200 -4.022159   POINT (39.622 -4.022159)     Mombasa   Changamwe
## 6       6 39.63788 -4.008725 POINT (39.63788 -4.008725)     Mombasa       Jomvu
##    admin2.name.full
## 1 Mombasa_Changamwe
## 2 Mombasa_Changamwe
## 3 Mombasa_Changamwe
## 4 Mombasa_Changamwe
## 5 Mombasa_Changamwe
## 6     Mombasa_Jomvu
```

```r
head(cluster.info$wrong.point)
```

```
## [1] 778
```

The following plots are histogram of the number of clusters sampled and the unweighted number of women who had a recent birth or stillbirth in each Admin-1 and Admin-2 areas in the 2022 Kenya DHS.

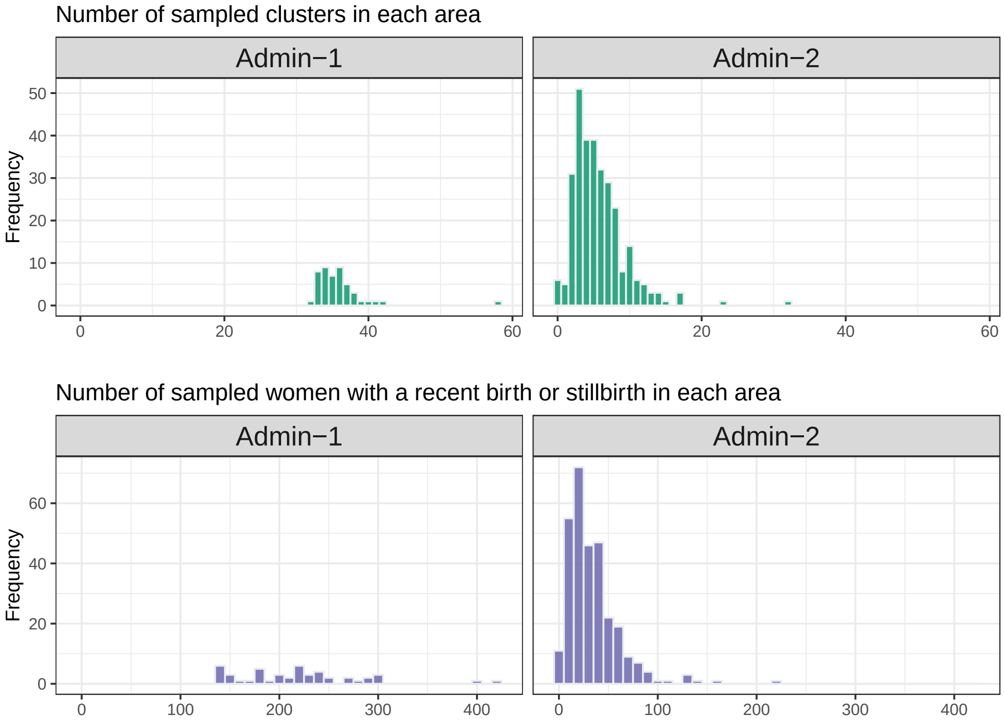

Figure 3: Histogram of the number of clusters sampled and the unweighted number of women who had a recent birth or stillbirth in each Admin-1 and Admin-2 areas.

2.4 Extract area-level population information

2.4.1 Population aggregating prevalence to upper level

We use `aggPopulation()` to aggregate pixel-level population data into both Admin-1 and Admin-2 levels. The `raster` is an aggregated TIFF file representing the population of female aged 15 to 49 in Kenya in 2022, downloaded from the WorldPop site[2].

```
raster <- raster("../WorkflowPaper/Data/TIFF/KEN_population_v2_0_agesex_f15_49.tif")
raster_2019 <- raster("../WorkflowPaper/Data/TIFF/ken_ppp_2019.tif")
agg.pop1 <- aggPopulation(tiff = raster,
                          poly.adm = poly.adm1,
                          by.adm = "NAME_1",
                          fact = 10)
agg.pop2 <- aggPopulation(tiff = raster,
                          poly.adm = poly.adm2,
                          by.adm = "NAME_2",
                          by.adm.upper = "NAME_1",
                          fact = 10)
```

```
head(agg.pop1)
```

```
##        admin1.name population
## 1          Baringo   166182.9
## 2            Bomet   248754.0
## 3          Bungoma   524335.0
## 4            Busia   247421.9
## 5 Elgeyo-Marakwet   108171.3
```

[2]https://hub.worldpop.org/project/categories?id=8

```
## 6            Embu   186381.2
```

```r
head(agg.pop2)
```

```
##          admin2.name.full population     admin2.name admin1.name
## 1             Baringo_805    7083.26             805     Baringo
## 2 Baringo_Baringo Central   25104.92 Baringo Central     Baringo
## 3   Baringo_Baringo North   23507.06   Baringo North     Baringo
## 4   Baringo_Baringo South   25419.99   Baringo South     Baringo
## 5   Baringo_Eldama Ravine   33638.31   Eldama Ravine     Baringo
## 6         Baringo_Mogotio   16270.75         Mogotio     Baringo
```

2.4.2 Urban/rural fraction for stratification model

We use `getUR()` to obtain the urban/rural population and urban fraction at both the Admin-1 and Admin-2 levels. This step uses census population raster[3] for `tiff.pop`, the female population aged 15–49 raster for `tiff.survey`, and `urban.frac`, which has the Admin-1 urban fractions from the Kenya 2022 DHS final report. We also obtain `frac$UR_surface`, a raster where each pixel is coded as 1 for urban areas and 0 for rural areas.

```r
tableA <- read.csv("../WorkflowPaper/Data/tableA.csv")
tableA$County[tableA$County == "Nairobi City"] <- "Nairobi"
tableA$County[tableA$County == "Taita-Taveta"] <- "Taita Taveta"
tableA$County[tableA$County == "Homabay"] <- "Homa Bay"
urban.frac <- data.frame(admin1 = tableA$County,
                         frac = tableA$Urban / tableA$Total)
```

```r
head(urban.frac, n=5)
```

```
##       admin1      frac
## 1    Mombasa 1.0000000
## 2      Kwale 0.1430016
## 3     Kilifi 0.2690967
## 4 Tana River 0.2453954
## 5       Lamu 0.2754220
```

```r
frac <- surveyPrev::getUR(tiff.census = raster_2019,
                          tiff.survey = raster,
                          prop.census = urban.frac,
                          fact = 10,
                          poly.adm1 =poly.adm1,
                          poly.adm2 =poly.adm2,
                          varname1 = "NAME_1",
                          varname2 = "NAME_2")
```

```r
ad1<- SUMMER::mapPlot(data = frac$admin1.ur,
                     geo = poly.adm1,
                         by.data = "admin1.name",
                         by.geo = "NAME_1",
                         variable = "urban")
ad2<-  SUMMER::mapPlot(data = frac$admin2.ur,
                      geo = poly.adm2,
                          by.data = "admin2.name",
                          by.geo = "NAME_2",
                          variable = "urban")
```

[3] https://hub.worldpop.org/geodata/summary?id=6281

```r
ad1 | ad2
```

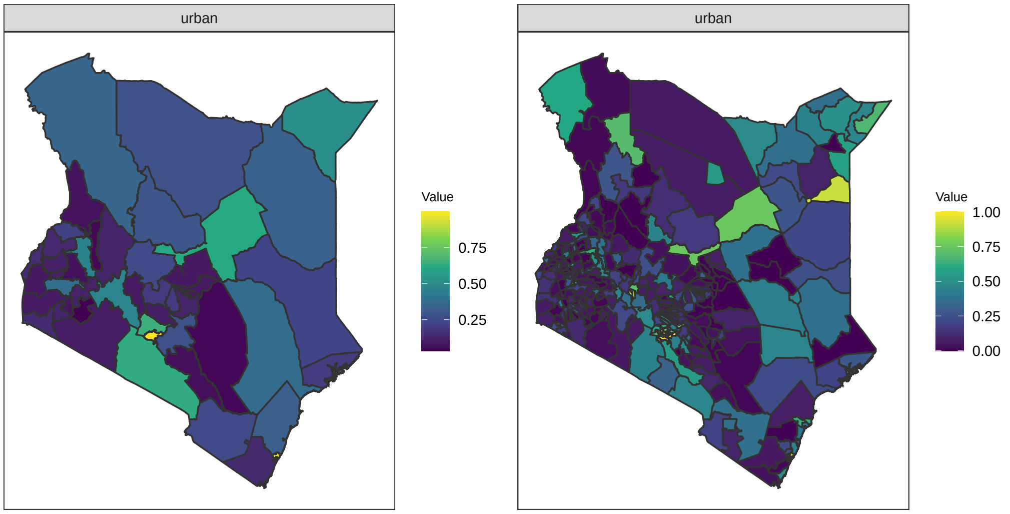

Figure 4: Admin-1 and Admin-2 urban fractions

We then use the `adminInfo()` function to extract a full list of names, population, and urban fractions at the corresponding administrative levels, and `admin.mat`, which contains the spatial adjacency matrix. `agg.pop1` and `proportion` refer to the administrative-level population and urban/rural fractions that we produced in the previous steps.

```r
admin.info1 <- surveyPrev::adminInfo(poly.adm = poly.adm1,
                                     admin = 1,
                                     by.adm = "NAME_1",
                                     agg.pop = agg.pop1,
                                     proportion = frac$admin1.ur)
admin.info2 <- surveyPrev::adminInfo(poly.adm = poly.adm2,
                                     admin = 2,
                                     by.adm = "NAME_2",
                                     by.adm.upper = "NAME_1",
                                     agg.pop =agg.pop2,
                                     proportion =  frac$admin2.ur)
```

```r
head(admin.info1$data,n=1)
```

```
##   admin1.name population     urban
## 1     Baringo   166182.9 0.1165844
```

```r
head(admin.info2$data,n=1)
```

```
##   admin1.name admin2.name admin2.name.full    urban population
## 1     Baringo         805      Baringo_805 0.166999    7083.26
##   population.admin1
## 1          166182.9
```

2.5 Cluster-level covariates

We use `getCovariate()` to extract covariates from a list of rasters into data frames at both the cluster-level and the pixel-level. The four covariates we use are census population, the total population, night time lights[4],

[4] https://ladsweb.modaps.eosdis.nasa.gov/missions-and-measurements/products/VNP46A4

vegetation index[5], and travel time to the nearest healthcare facility[6]. We set `standardize = TRUE` to scale the covariates.

```
avg.pop <- terra::rast(terra::aggregate(raster_2019, fact = 10, fun = sum,
                                         cores = parallel::detectCores()))
pop_survey <- terra::rast(terra::aggregate(raster, fact = 10, fun = sum,
                                         cores = parallel::detectCores()))
access_buffered <-  terra::rast('../WorkflowPaper/Data/TIFF/health_buffered.tif')
ndvi_ras_buffered <-  terra::rast('../WorkflowPaper/Data/TIFF/average_ndvi_buffered.tif')
light_ras_buffered <-  terra::rast('../WorkflowPaper/Data/TIFF/average_ntl_buffered.tif')

cov_raster_stack <- list(avg.pop=avg.pop,
                         access_to_health = access_buffered,
                         ndvi = ndvi_ras_buffered,
                         nighttime_light = light_ras_buffered)

kenya_cov <- surveyPrev::getCovariate(tiffs = cov_raster_stack,
                                      tiff.population = frac$pop_survey,
                                      UR.surface = frac$UR_surface,
                                      cluster.info = cluster.info,
                                      poly.adm = poly.adm2,
                                      by.adm = 'NAME_2',
                                      by.adm.upper = 'NAME_1',
                                      na.rm=TRUE,
                                      standardize = TRUE,
                                      fact = 1)
cov.uni=kenya_cov$cluster.cov[,c("cluster","avg.pop","access_to_health",
                                 "ndvi","nighttime_light")]
cov.pixel=kenya_cov$natl.grid[,c("admin2.name.full","admin1.name","strata",
                                 "Population","avg.pop","access_to_health",
                                 "ndvi","nighttime_light")]
```

```
head(cov.uni,n=1)
```

```
##   cluster  avg.pop access_to_health       ndvi nighttime_light
## 1       1 15.95546       -0.8777021 -0.2985815         52.6396
```

```
head(cov.pixel,n=1)
```

```
##   admin2.name.full admin1.name strata Population avg.pop access_to_health
## 1  Busia_Budalangi       Busia      0   16.69367       0       -0.2817005
##        ndvi nighttime_light
## 1 0.3652968      -0.0654404
```

```
kenya_cov$raster.stats
```

```
##               name         mean         sd
## 1          avg.pop   78.2222341  537.45033
## 2 access_to_health   78.1213813   88.17665
## 3             ndvi 3808.5595385 1728.08134
## 4  nighttime_light    0.6640658   10.14764
```

[5] https://lpdaac.usgs.gov/products/mod13a3v006/
[6] https://malariaatlas.org/project-resources/accessibility-to-healthcare/

3 Direct estimates

We use `directEST()` function to calculate direct estimates at Admin-1 and Admin-2 level. Summary statistics and samples are saved in `res.admin1` and `admin1_post`. When `aggregation = TRUE`, the output will also have `agg.natl` and `nation_post` at national level. Similar for Admin-2, we have `res.admin2` and `admin1_post2`, when `aggregation = TRUE`, output will include `agg.admin1`,`admin1_post`, `agg.natl` and `nation_post` at Admin-1 and national levels.

```
res_ad1 <- surveyPrev::directEST(data = data,
                                 cluster.info = cluster.info,
                                 admin = 1,
                                 aggregation = TRUE,
                                 admin.info = admin.info1)
res_ad2 <- surveyPrev::directEST(data = data,
                                 cluster.info = cluster.info,
                                 admin = 2,
                                 aggregation = TRUE,
                                 admin.info = admin.info2)
```

The direct estimates at both Admin-1 and Admin-2 levels are visualized in the map below.

```
poly.adm2$admin2.name.full <- paste0(poly.adm2$NAME_1, "_", poly.adm2$NAME_2)
out1<-res_ad1$res.admin1
out2<-res_ad2$res.admin2
g1<- SUMMER::mapPlot(out1,
                     geo = poly.adm1,
                         by.data = "admin1.name",
                         by.geo = "NAME_1",
                         variable = "direct.est")
g2<-  SUMMER::mapPlot(out2,
                      geo = poly.adm2,
                          by.data = "admin2.name.full",
                          by.geo = "admin2.name.full",
                          variable = "direct.est")
g1 | g2
```

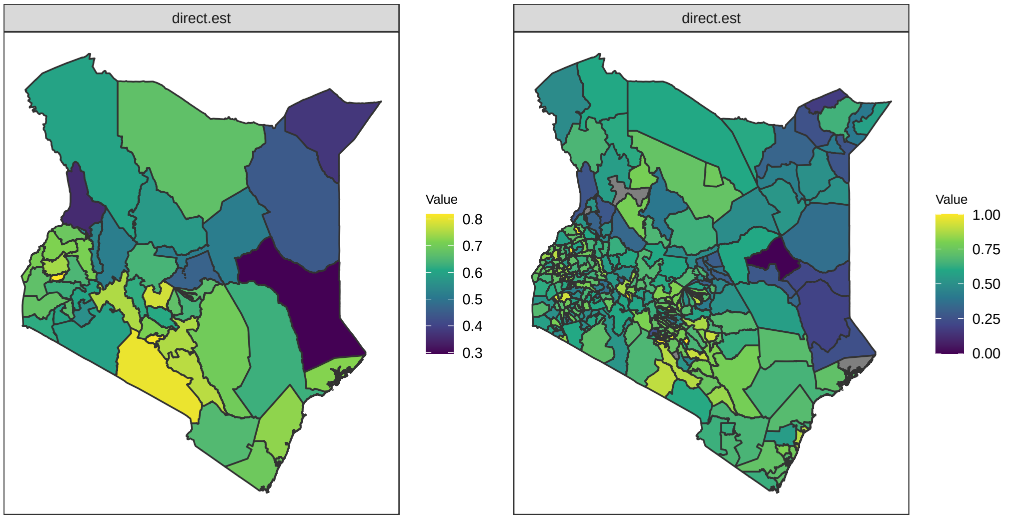

Figure 5: Admin-1 and Admin-2 Direct estimates

4 Area-level (Fay-Herriot) Estimates

4.1 Admin-1 Fay-Herriot estimates

We use `fhModel()` to get spatial Fay-Herriot estimates at different administrative levels. We choose `model = "bym2"` to use BYM2 as the random effect model, one can also use i.i.d. random effects by setting `model = "iid"`.

```
smth_res_ad1_bym2 <- surveyPrev::fhModel(data,
                                          cluster.info = cluster.info,
                                          admin.info = admin.info1,
                                          admin = 1,
                                          model = "bym2",
                                          aggregation = TRUE)
```

The Admin-1 Fay-Herriot model and its comparison with direct estimates are visualized below.

```
out1<-smth_res_ad1_bym2$res.admin1
names(out1)[names(out1) == "mean"] <- "Fay-Herriot estimates"
g1<- SUMMER::mapPlot(data = out1,
                     geo = poly.adm1,
                         by.data = "admin1.name",
                         by.geo = "NAME_1",
                         variable = "Fay-Herriot estimates")
g2<- surveyPrev::scatterPlot(res1=res_ad1$res.admin1,
                             res2=smth_res_ad1_bym2$res.admin1,
                             value1="direct.est",
                             value2="mean",
                             by.res1="admin1.name",
                             by.res2="admin1.name",
                             title="Admin-1 Estimates",
                             label1="Direct Estimates",
                             label2="Fay-Herriot estimates")

g1 | g2
```

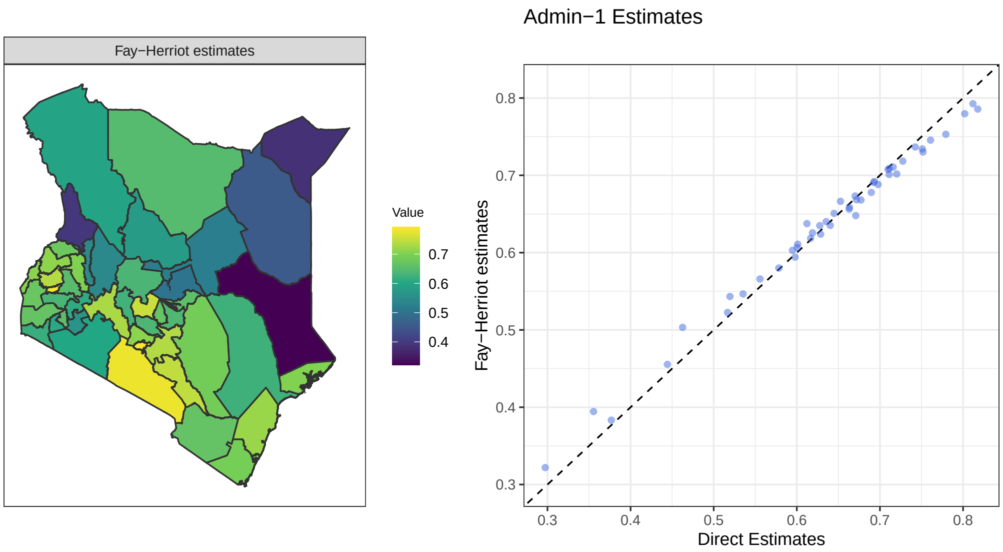

Figure 6: Map for Admin-1 Fay-Herriot estimates and comparison to direct estimates

4.2 Admin-2 Fay-Herriot estimates

For Admin-2, we set `var.fix = TRUE` in `fhModel()` to adjust the design-based variances used in Fay-Herriot model that are zero or close to zero, as described in our main paper.

```
FH_adm2_fix <- surveyPrev::fhModel(data = data,
                                   cluster.info = cluster.info,
                                   admin.info = admin.info2,
                                   admin = 2,
                                   model = "bym2",
                                   aggregation = TRUE,
                                   var.fix = TRUE)
```

The Admin-2 Fay-Herriot model and its comparison with direct estimates are visualized below.

```
out1<-FH_adm2_fix$res.admin2
names(out1)[names(out1) == "mean"] <- "Fay-Herriot estimates"
g1<- SUMMER::mapPlot(data = out1,
                     geo = poly.adm2,
                         by.data = "admin2.name.full",
                     by.geo = "admin2.name.full",
                     variable = "Fay-Herriot estimates")
g2<- surveyPrev::scatterPlot(res1=res_ad2$res.admin2,
                             res2=FH_adm2_fix$res.admin2,
                             value1="direct.est",
                             value2="mean",
                             by.res1="admin2.name.full",
                             by.res2="admin2.name.full",
                             title="Admin-2 Estimates",
                             label1="Direct Estimates",
                             label2="Fay-Herriot estimates")
g1 | g2
```

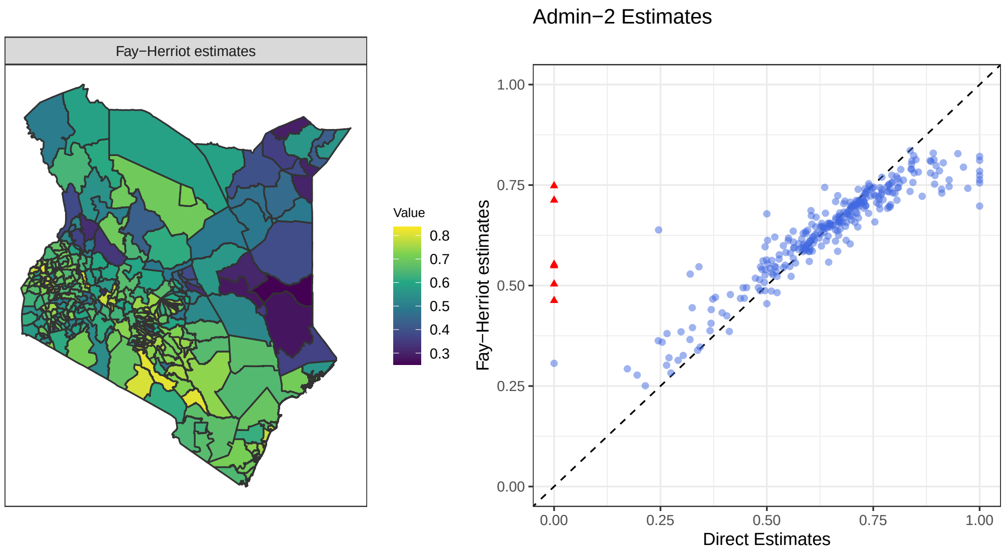

Figure 7: Map for Admin-2 Fay-Herriot estimates and comparison to direct estimates

4.3 Fay-Herriot model with area-level covariates

We now fit a Fay-Herriot model with area-level covariates. For Admin-1, `X = cov.adm1`, which includes population, night time lights, vegetation index, and travel time to the nearest healthcare facility at Admin-1 level. Similarly, `cov.adm2` contains the four covariates at Admin-2.

```
cov.adm1 <- read.csv("../WorkflowPaper/Data/covariate/Kenya_adm1_cov.csv")
for(j in 2:dim(cov.adm1)[2]) cov.adm1[, j] <- (cov.adm1[, j] - mean(cov.adm1[, j]))/ sd(cov.adm1[, j])
cov.adm2 <- read.csv("../WorkflowPaper/Data/covariate/Kenya_adm2_cov.csv")
for(j in 2:dim(cov.adm2)[2]) cov.adm2[, j] <- (cov.adm2[, j] - mean(cov.adm2[, j]))/ sd(cov.adm2[, j])
```

```
head(cov.adm1,n=3)
```

```
##   admin1.name   total_pop wt_night_time_light   wt_ndvi wt_health_access
## 1     Baringo -0.48489572          -0.3466018 0.1389989        0.4821602
## 2       Bomet -0.09146215          -0.3390252 1.2763054       -0.6051329
## 3     Bungoma  1.22455467          -0.3051305 0.5852044       -0.5447820
```

```
head(cov.adm2,n=3)
```

```
##          admin2.name.full  total_pop wt_night_time_light    wt_ndvi
## 1             Baringo_805 -2.1220246          -0.3734450 -0.3956606
## 2 Baringo_Baringo Central -0.7692442          -0.3299752  0.6793561
## 3   Baringo_Baringo North -0.6358879          -0.3760008  0.4137235
##   wt_health_access
## 1       -0.2780376
## 2       -0.2210590
## 3        0.1050781
```

Model fitting proceeds similar as before.

```
FH_adm1_cov <- surveyPrev::fhModel(data = data,
                                   X = cov.adm1,
                                   cluster.info = cluster.info,
                                   admin.info = admin.info1,
                                   admin = 1,
                                   model = "bym2",
                                   aggregation = FALSE)
FH_adm2_cov_fix <- surveyPrev::fhModel(data = data,
                                   X = cov.adm2,
                                   cluster.info = cluster.info,
                                   admin.info = admin.info2,
                                   admin = 2,
                                   model = "bym2",
                                   aggregation = FALSE,
                                   var.fix = TRUE)
```

```
g1<-surveyPrev::scatterPlot(res1=res_ad1$res.admin1,
                            res2=FH_adm1_cov$res.admin1,
                            value1="direct.est",
                            value2="mean",
                            by.res1="admin1.name",
                            by.res2="admin1.name",
                            title="Admin-1 Estimates",
                            label1="Direct Estimates",
                            label2="Fay-Herriot with area-level covariates")
g2<- surveyPrev::scatterPlot(res1=res_ad2$res.admin2,
```

```
                                    res2=FH_adm2_cov_fix$res.admin2,
                                    value1="direct.est",
                                    value2="mean",
                                    by.res1="admin2.name.full",
                                    by.res2="admin2.name.full",
                                    title="Admin-2 Estimates",
                                    label1="Direct Estimates",
                                    label2="Fay-Herriot with area-level covariates")
g1 | g2
```

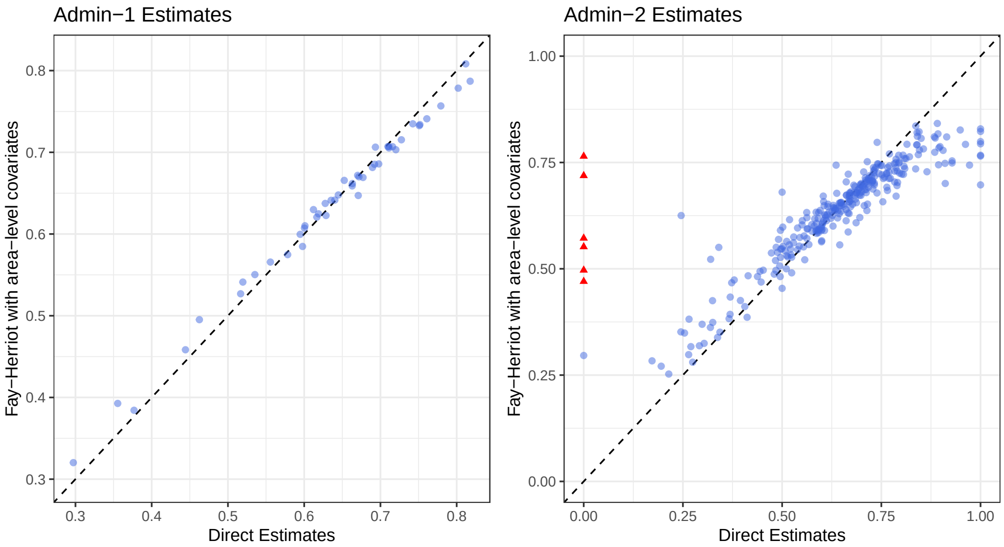

Figure 8: Comparisons of Fay-Herriot with area-level covariates to direct estimate

5 Cluster-Level Estimates

5.1 Urban/rural stratification

We first fit the unstratified model in both Admin-1 and Admin-2 below using `clusterModel()` by setting `stratification = FALSE`.

```
unit_adm1 <- surveyPrev::clusterModel(data=data,
                                      cluster.info=cluster.info,
                                      admin.info  = admin.info1,
                                      stratification = FALSE,
                                      model = "bym2",
                                      admin = 1,
                                      aggregation = TRUE,
                                      CI = 0.95)
unit_adm2 <- surveyPrev::clusterModel(data=data,
                                      cluster.info= cluster.info,
                                      admin.info = admin.info2,
                                      model = "bym2",
                                      stratification = FALSE,
                                      admin = 2,
                                      aggregation = TRUE,
                                      CI = 0.95)
```

The comparisons with direct estimates are visualized below.

```
g1<- surveyPrev::scatterPlot(res1=res_ad1$res.admin1,
                             res2=unit_adm1$res.admin1,
                             value1="direct.est",
                             value2="mean",
                             by.res1="admin1.name",
                             by.res2="admin1.name",
                             title="Admin-1 Estimates",
                             label1="Direct Estimates",
                             label2="Unstratified cluster-level model")
g2<- surveyPrev::scatterPlot(res1=res_ad2$res.admin2,
                             res2=unit_adm2$res.admin2,
                             value1="direct.est",
                             value2="mean",
                             by.res1="admin2.name.full",
                             by.res2="admin2.name.full",
                             title="Admin-2 Estimates",
                             label1="Direct Estimates",
                             label2="Unstratified cluster-level model")
g1 | g2
```

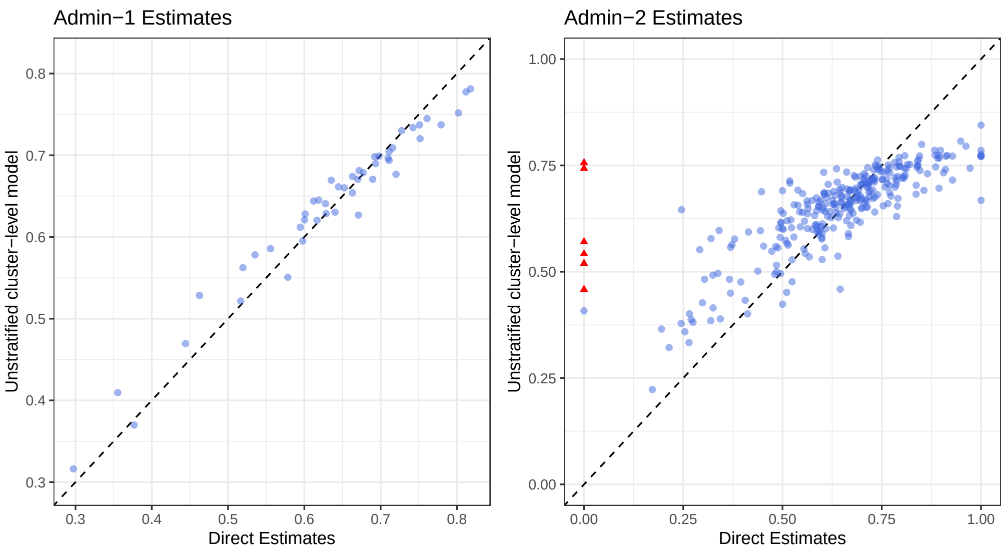

Figure 9: Comparisons of unstratified model to direct estimate

We then include the urban-rural stratification by setting `stratification = TRUE`. For Admin-2, The posterior summaries are stored in `res.admin2` with the stratification type (`"urban"`, `"rural"`, or `"full"`) identified in the `type` column of `res.admin2`. Corresponding posterior samples are available in `urban_post`, `rural_post` and `admin2_post`.

```
strata_adm1<- surveyPrev::clusterModel(data=data,
                                       cluster.info=cluster.info,
                                       admin.info  = admin.info1,
                                       stratification = TRUE,
                                       model = "bym2",
                                       admin = 1,
                                       aggregation = TRUE,
```

```r
                                              CI = 0.95)
strat_adm2 <- surveyPrev::clusterModel(data=data,
                                       cluster.info= cluster.info,
                                       admin.info = admin.info2,
                                       model = "bym2",
                                       stratification = TRUE,
                                       admin = 2,
                                       aggregation = TRUE,
                                       CI = 0.95)
```

The comparisons with direct estimates are visualized below.

```r
g1<-surveyPrev::scatterPlot(res1=res_ad1$res.admin1,
                            res2=subset(strata_adm1$res.admin1, type == "full"),
                            value1="direct.est",
                            value2="mean",
                            by.res1="admin1.name",
                            by.res2="admin1.name",
                            title="Admin-1 Estimates",
                            label1="Direct Estimates",
                            label2="Stratified cluster-level model")
g2<- surveyPrev::scatterPlot(res1=res_ad2$res.admin2,
                             res2=subset(strat_adm2$res.admin2, type == "full"),
                             value1="direct.est",
                             value2="mean",
                             by.res1="admin2.name.full",
                             by.res2="admin2.name.full",
                             title="Admin-2 Estimates",
                             label1="Direct Estimates",
                             label2="Stratified cluster-level model")

g1 | g2
```

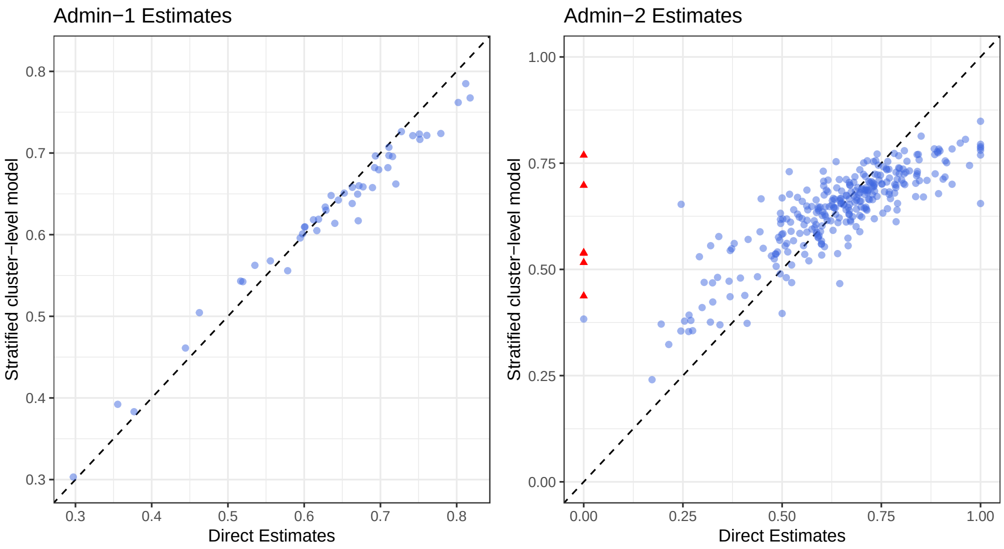

Figure 10: Comparisons of stratified model to direct estimate

5.2 Nested Admin-1 fixed effect

For the Admin-2 model, we include Admin-1 membership as a fixed effect by setting `nested = TRUE` in both the stratified and unstratified models.

```
nest_adm2 <- surveyPrev::clusterModel(data=data,
                                      cluster.info= cluster.info,
                                      admin.info = admin.info2,
                                      stratification = FALSE,
                                      nested = TRUE,
                                      model = "bym2",
                                      admin = 2,
                                      aggregation = TRUE,
                                      CI = 0.95)
strat_nest_adm2 <- surveyPrev::clusterModel(data=data,
                                            cluster.info=cluster.info,
                                            admin.info = admin.info2,
                                            stratification = TRUE,
                                            nested = TRUE,
                                            model = "bym2",
                                            admin = 2,
                                            aggregation = TRUE,
                                            CI = 0.95)
```

We also fit a model with an interaction between urban/rural and Admin-1 by setting `interact = TRUE` in addition to `nested = TRUE` and `stratification = TRUE`.

```
interact_adm2 <- surveyPrev::clusterModel(data=data,
                                 cluster.info=cluster.info,
                                 admin.info = admin.info2,
                                 stratification = TRUE,
                                 nested = TRUE,
                                 interact = TRUE,
                                 model = "bym2",
                                 admin = 2,
                                 aggregation = TRUE,
                                 CI = 0.95)
```

The comparisons with direct estimates are visualized below.

```
g1<- surveyPrev::scatterPlot(res1=res_ad2$res.admin2,
                             res2=nest_adm2$res.admin2,
                             value1="direct.est",
                             value2="mean",
                             by.res1="admin2.name.full",
                             by.res2="admin2.name.full",
                             title="Unstratified nested",
                             label1="Direct Estimates",
                             label2="cluster-level model")
g2<- surveyPrev::scatterPlot(res1=res_ad2$res.admin2,
                        res2=subset(strat_nest_adm2$res.admin2, type == "full"),
                        value1="direct.est",
                        value2="mean",
                        by.res1="admin2.name.full",
                        by.res2="admin2.name.full",
                        title="Stratified nested",
```

```
                             label1="Direct Estimates",
                             label2="Cluster-level model")
g3<- surveyPrev::scatterPlot(res1=res_ad2$res.admin2,
                             res2=subset(interact_adm2$res.admin2, type == "full"),
                             value1="direct.est",
                             value2="mean",
                             by.res1="admin2.name.full",
                             by.res2="admin2.name.full",
                             title="With interaction",
                             label1="Direct Estimates",
                             label2="Cluster-level model")
g1 | g2 |g3
```

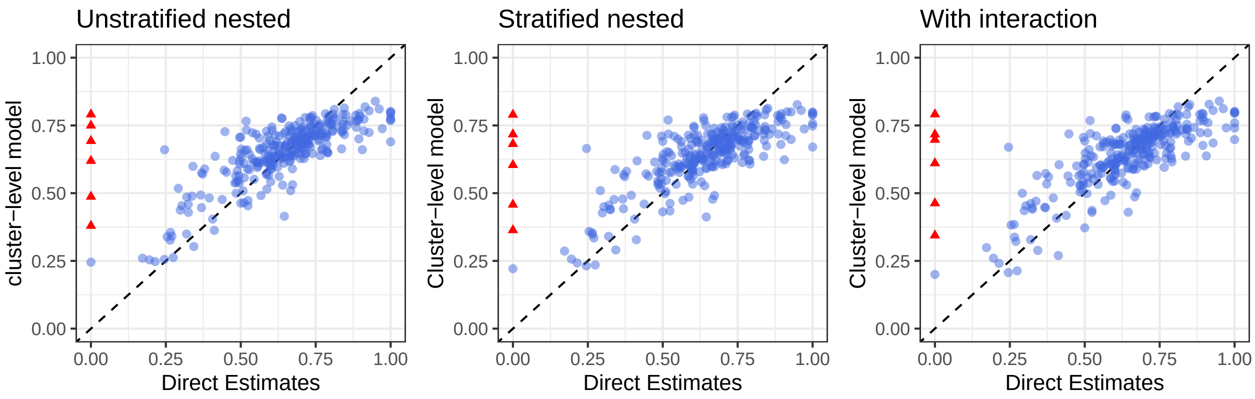

Figure 11: Comparisons of nested cluster-level models to direct estimate

5.2.1 Selecting fixed effect parameterization

In terms of model selection, we run a likelihood ratio test on the interaction term between urban/rural and Admin-1. The resulting p-value is 0.49, suggesting that the interaction term is not statistically significant.

```
design<-survey::svydesign(id=~cluster+householdID,
                          weights=~weight,
                          data=data)
svyglm_model<-survey::svyglm(value ~ strata*v024,
                             design=design,
                             family=quasibinomial())
test<-survey::regTermTest(svyglm_model,
                          ~strata:v024,
                          method="LRT")
test$p
```

```
## [1] 0.4920784
```

This finding is also supported by the WAIC from the cluster-level model results. Among the five fitted cluster-level models, the stratified nested model has the smallest WAIC.

```
waicc=c(unit_adm2$inla$waic$waic,
        strat_adm2$inla$waic$waic,
        nest_adm2$inla$waic$waic,
        strat_nest_adm2$inla$waic$waic,
        interact_adm2$inla$waic$waic)
waiccc=data.frame( model=c("Unstratified Cluster-Level Model",
```

```r
                  "Stratified Cluster-Level Model",
                  "Unstratified Nested Cluster-Level Model",
                  "Stratified Nested Cluster-Level Model",
                  "Stratified Nested with Interaction Cluster-Level Model"),
                   waic=waicc)
waiccc_sorted <- waiccc[order(waiccc$waic), ]
kable(waiccc_sorted,
      digits = 3,
      caption = "WAIC Comparison Table",
      row.names = FALSE,
      booktabs = TRUE) %>%
  kable_styling(latex_options = c("HOLD_position", "striped", "bordered"))
```

Table 1: WAIC Comparison Table

model	waic
Stratified Nested Cluster-Level Model	5266.385
Stratified Cluster-Level Model	5275.214
Stratified Nested with Interaction Cluster-Level Model	5298.001
Unstratified Nested Cluster-Level Model	5324.165
Unstratified Cluster-Level Model	5328.458

5.3 Using covariates

5.3.1 Area-level covariates

Similar to the Fay-Herriot model with area-level covariates, we fit cluster-level models using area-level covariates, with `X = cov.adm1` or `X = cov.adm2` for the Admin-1 and Admin-2 models accordingly.

```r
strata_adm1_area <- surveyPrev::clusterModel(data=data,
                                             X=cov.adm1,
                                             cluster.info=cluster.info,
                                             admin.info = admin.info1,
                                             stratification = TRUE,
                                             model = "bym2",
                                             admin = 1,
                                             aggregation = TRUE,
                                             CI = 0.95)
strat_nest_adm2_area <- surveyPrev::clusterModel(data=data,
                                                 X=cov.adm2,
                                                 cluster.info=cluster.info,
                                                 admin.info = admin.info2,
                                                 stratification = TRUE,
                                                 nested = TRUE,
                                                 interact = FALSE,
                                                 model = "bym2",
                                                 admin = 2,
                                                 aggregation = TRUE,
                                                 CI = 0.95)
```

The comparisons with direct estimates are visualized below.

```
g1<-surveyPrev::scatterPlot(
                res1=subset(strata_adm1$res.admin1, type == "full"),
                res2=subset(strata_adm1_area$res.admin1, type == "full"),
                value1="mean",
                value2="mean",
                by.res1="admin1.name",
                by.res2="admin1.name",
                title="Admin-1 Stratified cluster-level model",
                label1="Without area-level covariates",
                label2="With area-level covariates")
g2<-surveyPrev::scatterPlot(
                res1=subset(strat_nest_adm2$res.admin2, type == "full"),
                res2=subset(strat_nest_adm2_area$res.admin2, type == "full"),
                value1="mean",
                value2="mean",
                by.res1="admin2.name.full",
                by.res2="admin2.name.full",
                title="Admin-2 Stratified nested cluster-level model",
                label1="Without area-level covariates",
                label2="With area-level covariates")
g1 | g2
```

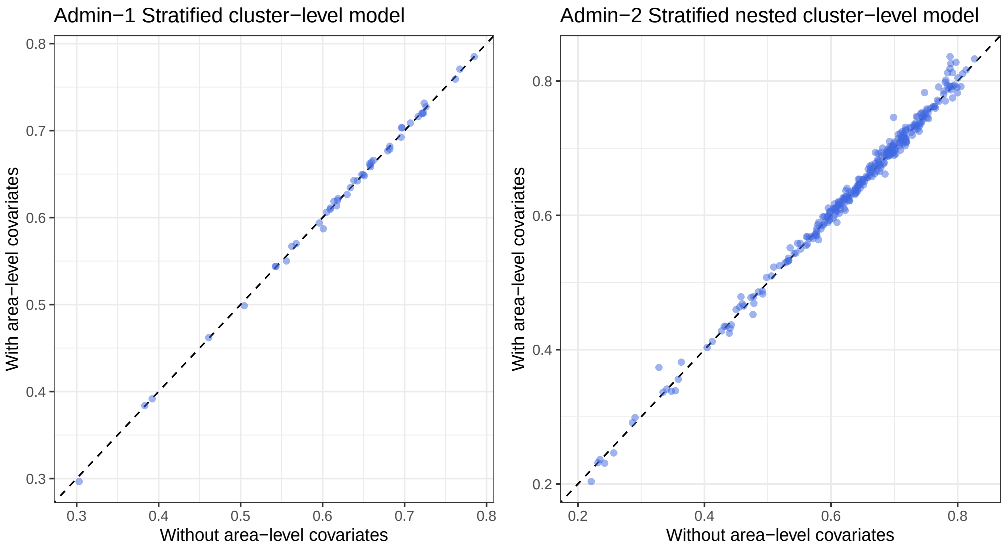

Figure 12: Comparisons of cluster-level model with and without area-level covariates

5.3.2 cluster-level covariates

We then fit cluster-level model with cluster-level covariates by providing `X.unit=cov.uni` for modeling and `X.pixel=cov.pixel` for aggregation. Both `cov.uni` and `cov.pixel` are produced using `surveyPrev::getCovariate()` (see Section 2.5).

Since the aggregation is based on pixel-level population and strata, rather than the area-level population and urban fractions in `admin.info`, the posterior summaries for Admin-2, Admin-1, and national levels are provided in `agg.admin2`, `agg.admin1`, and `agg.admin0`, respectively, for both Admin-1 and Admin-2 models, even without setting `aggregation = TRUE`. Posterior samples at different administrative levels, as well as for urban, rural, are also provided.

```r
strata_adm1_unit <- surveyPrev::clusterModel(data=data,
                                X.unit=cov.uni,
                                X.pixel=cov.pixel,
                                cluster.info=cluster.info,
                                admin.info = admin.info1,
                                stratification = TRUE,
                                model = "bym2",
                                admin = 1,
                                CI = 0.95)
strat_nest_adm2_unit<- surveyPrev::clusterModel(data=data,
                                   X.unit=cov.uni,
                                   X.pixel=cov.pixel,
                                   cluster.info=cluster.info,
                                   admin.info = admin.info2,
                                   stratification = TRUE,
                                   nested = TRUE,
                                   interact = FALSE,
                                   model = "bym2",
                                   admin = 2,
                                   CI = 0.95)
```

The comparisons with direct estimates are visualized below.

```r
g1<- surveyPrev::scatterPlot(
                 res1=subset(strata_adm1$res.admin1, type == "full"),
                 res2=subset(strata_adm1_unit$agg.admin1, type == "full"),
                 value1="mean",
                 value2="mean",
                 by.res1="admin1.name",
                 by.res2="admin1.name",
                 title="Admin-1 Stratified cluster-level model",
                 label1="Without cluster-level covariates",
                 label2="With cluster-level covariates")
g2<- surveyPrev::scatterPlot(
                 res1=subset(strat_nest_adm2$res.admin2, type == "full"),
                 res2=subset(strat_nest_adm2_unit$agg.admin2, type == "full"),
                 value1="mean",
                 value2="mean",
                 by.res1="admin2.name.full",
                 by.res2="admin2.name.full",
                 title="Admin-2 Stratified nested cluster-level model",
                 label1="Without cluster-level covariates",
                 label2="With cluster-level covariates")
g1 | g2
```

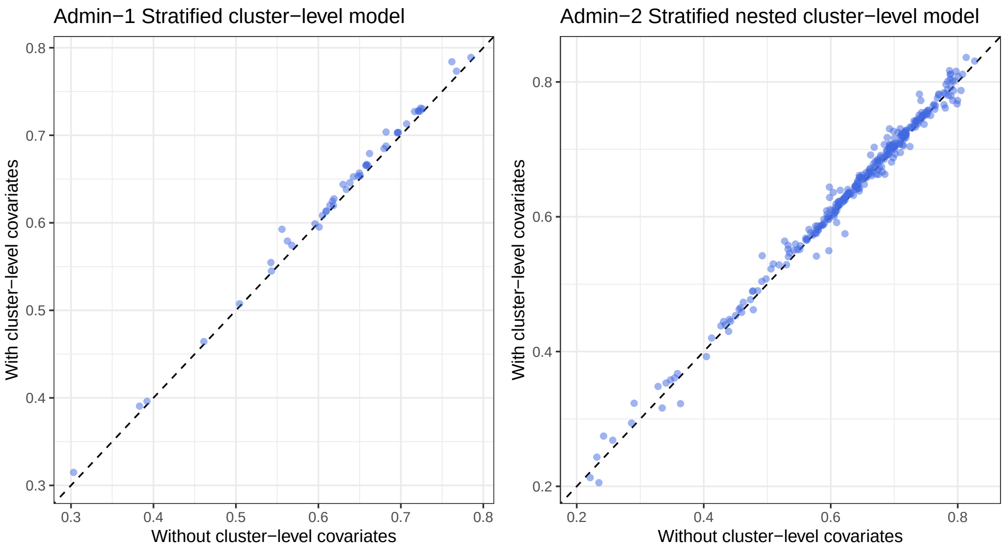

Figure 13: Comparisons of cluster-level model with and without cluster-level covariates

Finally, we compare the Admin-1 and Admin-2 models with area-level and cluster-level covariates below.

```
out1 <- subset(strata_adm1_area$res.admin1, type == "full")[,
                              c("admin1.name", "mean", "cv", "lower", "upper")]
out1$model <- "Admin-1: With area-level covariates"
out2 <- subset(strata_adm1_unit$agg.admin1, type == "full")[,
                              c("admin1.name", "mean", "cv", "lower", "upper")]
out2$model <- "Admin-1: With cluster-level covariates"
out <- rbind(out1, out2)
g1 <- mapPlot(data = out,
              geo = poly.adm1,
              by.data = "admin1.name",
              by.geo = "NAME_1",
              is.long = TRUE,
              variable = "model",
              value = "mean",
              legend.label = "Mean",
              direction = -1,
              ncol = 2,
              size = .05,
              border = "gray50")
out1 <-subset(strat_nest_adm2_area$res.admin2,type == "full")[,
               c("admin2.name.full", "mean", "lower", "upper")]
out1$model <- "Admin-2: With area-level covariates"
out2 <- subset(strat_nest_adm2_unit$agg.admin2,type == "full")[,
               c("admin2.name.full", "mean", "lower", "upper")]
out2$model <-  "Admin-2: With cluster-level covariates"
out <- rbind(out1, out2)
out$model <- factor(out$model, levels = unique(out$model))
out$width <- out$mean - out$lower
g2 <- mapPlot(data = out,
              geo = poly.adm2,
              by.data = "admin2.name.full",
              by.geo = "admin2.name.full",
```

```
                 is.long = TRUE,
                 variable = "model",
                 value = "mean",
                 legend.label = "Mean",
                 direction = -1,
                 ncol = 4,
                 size = .05,
                 border = "gray50")
g1 / g2
```

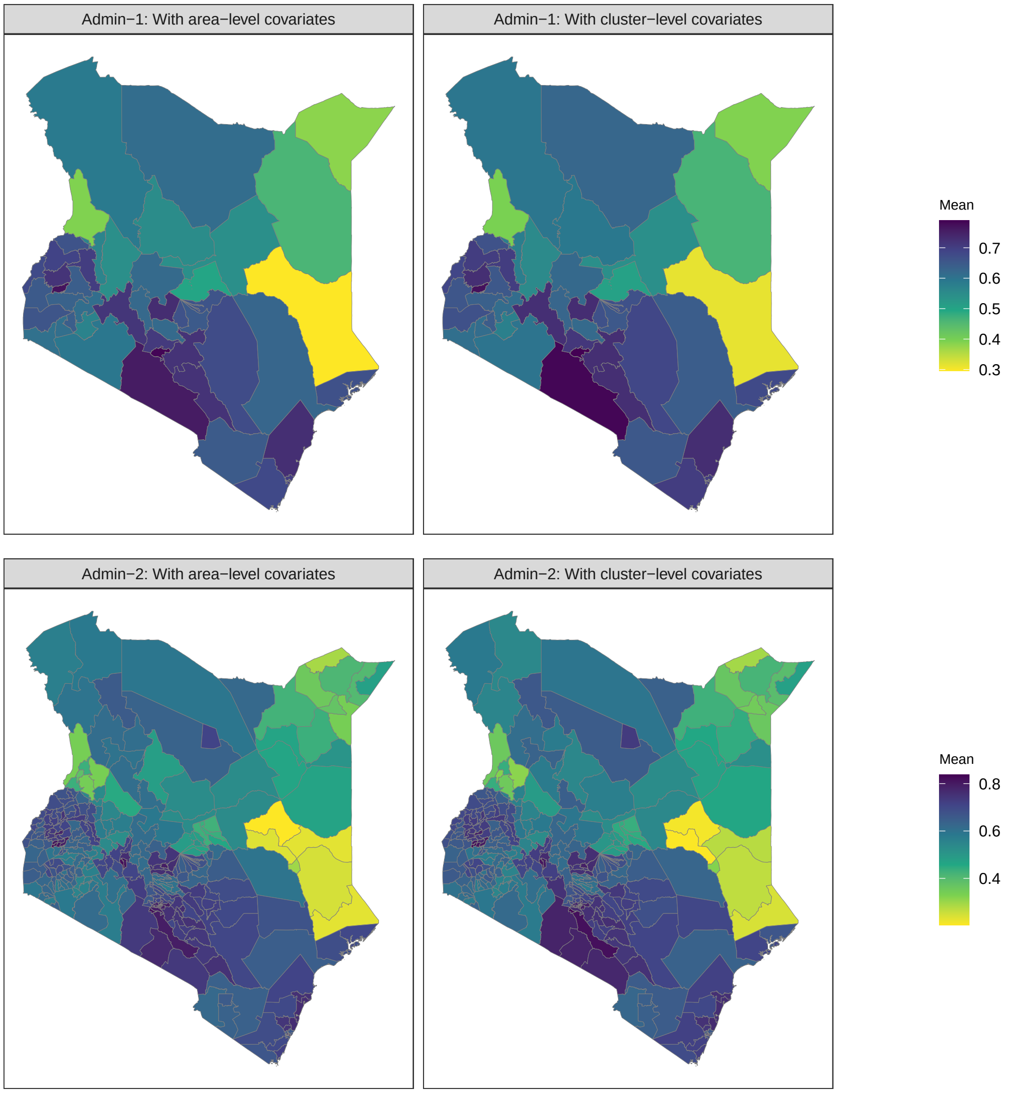

Figure 14: Maps for stratified nested cluster-level models with area-level covariates and with cluster-level covariates

6 Visualization

We use `SUMMER::ridgePlot()` to show the posterior marginal distributions of the Admin-1 stratified model results across regions.

```r
v1 <- SUMMER::ridgePlot(x = strata_adm1, direction = -1) +
        theme_bw() +
        theme(legend.position = "none")
v1
```

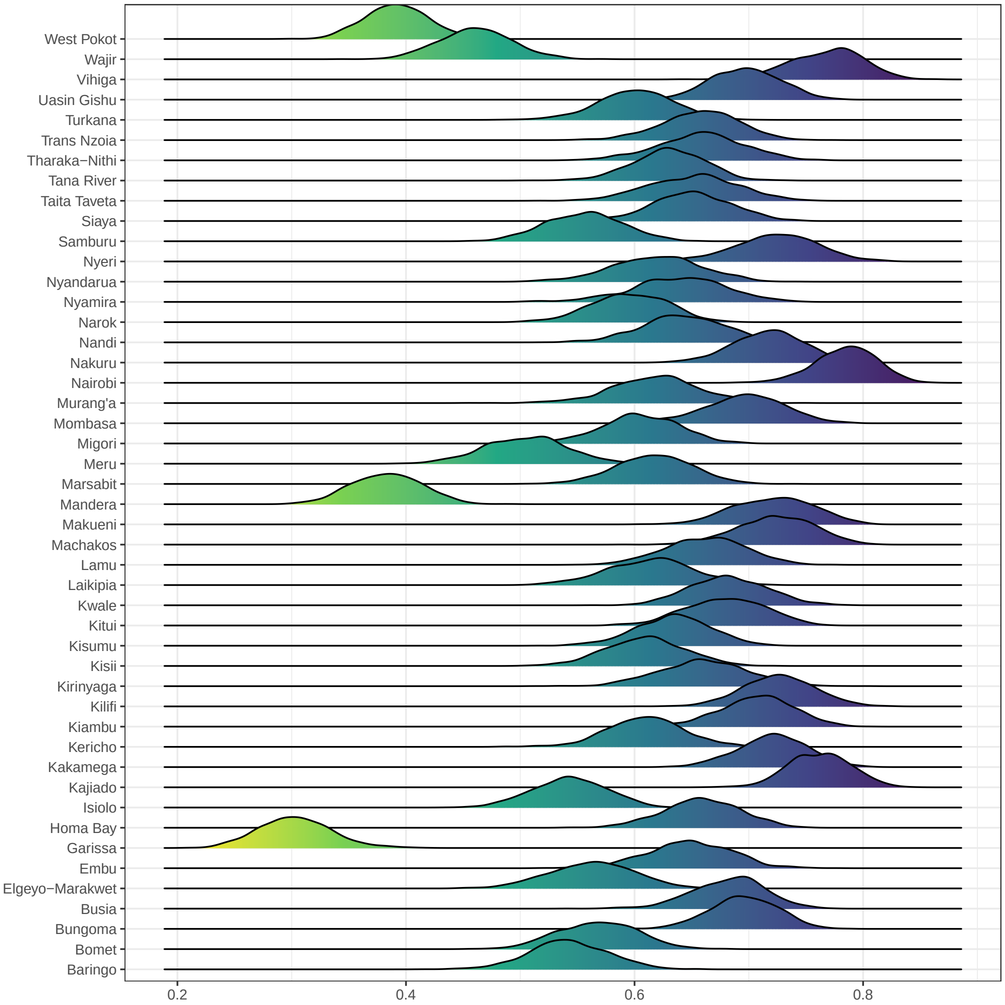

Figure 15: Ridge plot of the posterior distributions of the Admin-1 prevalence, based on the stratified cluster-level model.

We use `surveyPrev::rankPlot()` to visualize the rank of posterior marginal distributions from the Admin-1 stratified model results.

```r
v2 <- surveyPrev::rankPlot(x = strata_adm1,direction = 1)+
        ggtitle("Rank Plot: Admin-1 stratified cluster-level Model")+
        theme_bw() +
        theme(legend.position = "none")
v2
```

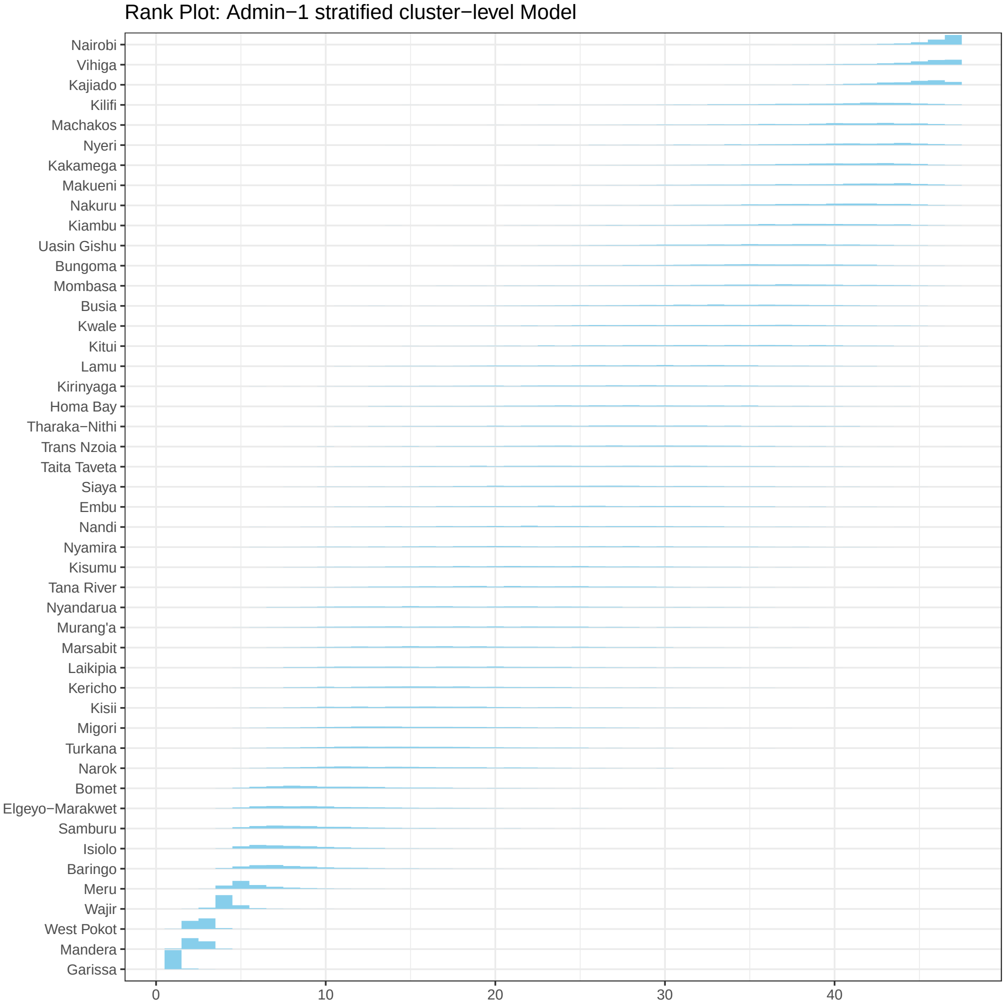

Figure 16: Rank plot of the posterior distributions of the Admin-1 prevalence, based on the stratified cluster-level model.

We use `surveyPrev:: exceedPlot` to show the probability that prevalence exceeds the threshold 70% for both Admin-1 stratified cluster-level model and Admin-2 stratified nested cluster-level model.

```
thres <- .7
legend <- paste0("Probability\nExceeding ", thres*100, "%\n")
v3 <- surveyPrev::exceedPlot(strata_adm1,
                  threshold = thres,
                  exceed = TRUE,
                  direction = -1,
                  border = "gray80",
                  size = 0,
                  geo = poly.adm1,
                  by.geo = "NAME_1",
                  ylim = c(0, 1)) +
  scale_fill_distiller(legend, palette = "Spectral", direction = 1) +
  ggtitle("Admin 1 Exceedance Probability")
v4 <- surveyPrev::exceedPlot(strat_nest_adm2,
                  threshold = thres,
                  exceed = TRUE,
```

```
                    direction = -1,
                    border = "gray80",
                    size = 0,
                    geo = poly.adm2,
                    by.geo = "admin2.name.full", ylim = c(0, 1)) +
  scale_fill_distiller(legend, palette = "Spectral", direction = 1) +
  ggtitle("Admin 2 Exceedance Probability")

v3 | v4
```

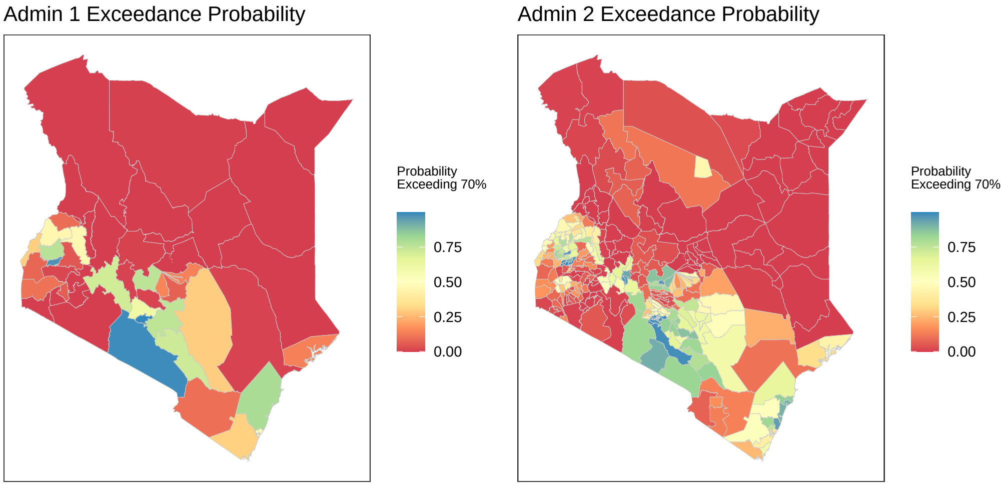

Figure 17: Exceedance probability plot, based on Admin-1 stratified cluster-level model and Admin-2 stratified nested cluster-level model

Rue, Håvard, Sara Martino, and Nicolas Chopin. 2009. "Approximate Bayesian Inference for Latent Gaussian Models by Using Integrated Nested Laplace Approximations." *Journal of the Royal Statistical Society Series B: Statistical Methodology* 71 (2): 319–92.
